# Short and long-term metabolic outcomes in patients with type 1 and type 2 diabetes receiving a simultaneous pancreas kidney allograft

**DOI:** 10.1186/s12902-020-0506-9

**Published:** 2020-02-27

**Authors:** Hans-Michael Hau, Nora Jahn, Maximilian Brunotte, Andri Arnosson Lederer, Elisabeth Sucher, Franz Maximilian Rasche, Daniel Seehofer, Robert Sucher

**Affiliations:** 1 Department of Visceral, Transplantation, Vascular and Thoracic Surgery, University Hospital of Leipzig, Liebigstrasse 20, 04103 Leipzig, Germany; 2 Department of Visceral, Transplantation, Vascular and Thoracic Surgery, University Hospital of Dresden, Fetcherstrasse 74, 01307 Dresden, Germany; 3 Department of Anesthesiology and Intensive Care Medicine, University Hospital of Leipzig, Liebigstrasse 20, 04103 Leipzig, Germany; 4 Department of Surgery, University Hospital of Leipzig, Liebigstrasse 20, 04103 Leipzig, Germany; 5 Department of Gastroenterology and Rheumatology, University Hospital of Leipzig, Liebigstrasse 20, 04103 Leipzig, Germany; 6 Department of Internal Medicine, Neurology, Dermatology, Clinic for Endocrinology, Diabetology and Nephrology, University Hospital of Leipzig, Liebigstrasse 20, 04103 Leipzig, Germany

**Keywords:** Pancreas transplantation, Type II diabetes, Endocrine and metabolic long-term results, Immunosuppression

## Abstract

**Background:**

In contrast to insulin-dependent type 1 diabetes mellitus (T1DM), the indication for Simultaneous pancreas-kidney transplantation (SPK) in patients with type 2 diabetes mellitus (T2DM) is still ambiguous and wisely Eurotransplant (ET) only granted transplant-permission in a selected group of patients. However, with regard to improvement of metabolic conditions SPK might still be a considerable treatment option for lean insulin dependent type 2 diabetics suffering from renal disease.

**Methods:**

Medical data (2001–2013) from all consecutive T1DM and T2DM patients who received a SPK or kidney transplant alone (KTA) at the University Hospital of Leipzig were analyzed. Donor, recipients and long-term endocrine, metabolic and graft outcomes were investigated for T1DM and T2DM-SPK recipients (transplanted upon a special request allocation by ET) and T2DM patients who received a KTA during the same period.

**Results:**

Eighty nine T1DM and 12 T2DM patients received a SPK and 26 T2DM patients received a KTA. Patient survival at 1 and 5 years was 89.9 and 88.8% for the T1DM group, 91.7 and 83.3% for the T2DM group, and 92.3 and 69.2% for the T2DM KTA group, respectively (*p < 0.01*). Actuarial pancreas graft survival for SPK recipients at 1 and 5 years was 83.1 and 78.7% for the T1DM group and 91.7 and 83.3% for the T2DM group, respectively (*p = 0.71*). Kidney allograft survival at 5 years was 79.8% for T1DM, 83.3% for T2DM, and 65.4% for T2DM KTA (*p < 0.01*). Delayed graft function (DGF) rate was significantly higher in type 2 diabetics received a KTA. Surgical, immunological and infectious complications showed similar results for T1DM and T2DM recipients after SPK transplant and KTA, respectively. With regard to the lipid profile, the mean high-density lipoprotein (HDL)- cholesterol levels were significantly higher in T1DM recipients compared to T2DM patients before transplantation (*p = 0.02*) and remained significantly during follow up period.

**Conclusion:**

Our data demonstrate that with regard to metabolic function a selected group of patients with T2DM benefit from SPK transplantation. Consensus guidelines and further studies for SPK transplant indications in T2DM patients are still warranted.

## Background

Type 1 and Type 2 diabetes mellitus are a heterogeneous group of diseases with an extensive variety and overlap in clinical presentation and disease progression [1]. Type 2 diabetes mellitus (T2DM), which accounts for 90% of all diabetes cases and 30% of end stage renal failure cases worldwide, plays a key role in the metabolic syndrome, which is a cluster of medical conditions that besides diabetes includes central obesity, high blood pressure and high triglyceride levels [2].

Simultaneous pancreas-kidney transplantation (SPK) with no hesitation must be considered as treatment of choice for patients with type 1 diabetes mellitus (T1DM) and end-stage renal disease (ESRD) [3, 4]. However, Type 2 diabetes mellitus (T2DM), which accounts for 90% of all diabetes cases worldwide, by comparison remains a disproportionally low 10% entity in all SPK performed in the Eurotransplant (ET) region [5–7]. This is due to the fact that the majority of T2DM patients might not benefit from SPK and should rather continue oral antidiabetic and antihypertensive medication [8].

The ideal T2DM patient scheduled for SPK is lean, insulin dependent and suffers from renal disease [7]. This is why ET introduced special allocation guidelines for patients with T2DM requesting for a SPK [6]. Few studies from European and international transplant centers report, that under these strict selection criteria medium- and long-term outcomes for T1DM and T2DM patients receiving pancreas transplants are comparable good [9–14]. Still, the popularity of pancreas transplantation for T2DM remains low. This is due to the fact that many patients might not easily fit into a single T1DM or T2DM category since numerous pathologic processes are involved in the development of diabetes and to date there are no reliable tests or diagnostic classifications which can precisely distinguish between both disease entities [7, 15, 16].

In search for a decent differentiation between T1DM and T2DM the American Diabetes Association (ADA) and the World Health Organisation (WHO) have published guidelines that should help classify patients as having T2DM [7, 17]. Although precise, these criteria lack crucial information on the subject’s family history and insulin-therapy start and dosage as well as data on islet and insulin autoantibodies.

Recently few studies were performed investigating the metabolic outcomes of pancreas transplantation in T1DM, however limited data are available for T2DM patients [10, 18–20]. First reports nonetheless indicate that pancreas transplantation is capable of sustaining favorable endocrine functions and that there is no significant difference in insulin resistance or b-cell function between T1DM and T2DM in the long term [10].

The purpose of this study was to examine short- and long-term effects with regard to metabolic control und beta cell function of T1DM und T2DM patients after SPK and T2DM patients of a kidney transplantation alone (KTA).

## Methods

### Study population

After approval by the local ethics committee [AZ: Nr: 111–16-14,032,016] medical data from all patients undergoing pancreas−/and kidney transplantation at the University Hospital of Leipzig between 2001 and 2013 were retrospectively analyzed from a prospectively collected data base. With regard to renal damage patients with multiple or other causes than diabetic kidney disease as the lead reason for renal damage were excluded from analysis. Patient and graft outcomes after transplantation were analyzed for T1DM and T2DM patients. 26 consecutive type II diabetic recipients of a kidney alone transplantation served as another demographic group.

Due to the national and international guidelines of Eurotransplant [ET] Region type II diabetics getting a special request allocation for transplantation. This special request status was approved by local committee of ET Pancreas Advisory Committee if some main criterions of defining diabetes mellitus as type II diabetes were fulfilled in these patients [6].

These ET conditions were applied in accordance with the guidelines of American Diabetes Association (ADA) and the World Health Organization (WHO) [7, 17]:

– Onset of diabetes mellitus at or after 40 years of age, no history of diabetic ketoacidosis, and 1 of the following
  - weight at diagnosis and/or maximum weight greater than 115% of the ideal body weight
  - Non consistent insulin therapy during the first 2 years after diabetes diagnosis.
2. Onset of diabetes from 30 to 39 years of age, no history of diabetic ketoacidosis and both 1a and 1b.

Selection criteria for pancreas transplantation in T2DM in our center include patients < 60 years with a body mass index of < 30 kg/m^2^, fasting C-peptid levels < 10 ng/ml, insulin requirement for a minimum of 5 years with daily requirements of less than 1 U/kg per day, absence of pancreatic antibodies (anti-glutamic acid decarboxylase (GAD)), islet cell antibodies (ICA), anti-tyrosine phosphotase (anti-IA2), absence of severe vascular disease and adequate cardiac function.

Patients designated as Type I Diabetes Mellitus included those with early onset of disease, insulin requirement from onset, and/or presence of diabetic ketoacidosis and presence of one or more pancreatic antibodies and C-peptide negativity.

Following demographic and clinicopathological data of the study population were collected and analyzed before, at the time of and after transplantation for each patient:

Pretransplant data including recipient and donor characteristics like age, gender, body mass index (BMI), donor cause of death. Further data are age of recipient at onset of diabetes mellitus, duration of diabetes mellitus, insulin amount, time of the waiting list, preformed human leukocyte antigen (HLA) antibodies and degree of HLA mismatch, duration of pretransplant dialysis, metabolic endocrine and lipid metabolism, secondary diabetic complications (retinopathy, nephropathy, neuropathy), information about the cardiovascular system like presence of arterial obstructive disease, coronary heart disease (coronary artery bypass graft (CABG)/stent), hypertension and number of antihypertensive drugs and blood pressure.

Collected peri-transplant and posttransplant data including cold ischemia time (CIT), immunosuppressive regimes, surgical and infectious complications, number of rejection episodes, delayed graft function pancreas and kidney such as kidney and metabolic endocrine metabolism at discharge.

Patient, pancreas and kidney graft function such as metabolic endocrine/lipid metabolism were analyzed up to 5 years post transplantation.

### SPK surgical technique

The procurement of pancreas and kidney allografts were described previously and were performed by international standards and guidelines [21, 22].

In short, for pancreas transplantation, the organ was placed intraperitoneally in the right iliac fossa. The arterial anastomosis was usually sutured to the recipient’s common iliac artery. The venous anastomosis was done by using the inferior caval vein of the recipient [23]. Drainage of the exocrine pancreatic secretions was performed by enteric drainage via a hand-sutured side-to-side duodenojejunostomy 40 cm beyond the flexure of Treitz [23, 24]. For kidney transplantation, we used a standard technique described previously [25]. In short, kidneys were placed into the contralateral iliacal fossa [25]. Vascular anastomoses were usually performed in an end-to-side technique to the recipient’s external or common iliacal vessels. The ureter was implanted into the bladder as an extravesical ureteroneocystostomy according to the Lich-Gregoir technique [25].

### Immunosuppression

Immunosuppression protocol of our center consisted of an induction therapy, maintenance immunosuppression with a calcineurininhibitor (CNI) (mostly tacrolimus), an antimetabolite (mostly mycophenolate mofetile (MMF) or sirolimus (SRL)) and steroids [26].

As standard induction therapy, patients usually received antithymocyte globulin (ATG) with an initial dose of 4 mg/kg body weight before transplantation and followed by 1–1.5 mg/kg body weight on postoperative days 1–3 [26].

In some cases, the interleukin-2 receptor antagonist basiliximab (20 mg) was used as induction therapy before transplantation and followed at postoperative day 4.

During months 1–3 after transplantation, tacrolimus target levels were 10–12 ng/ml, and 8–10 ng/ml during months 4 to 12. Following the protocol, tacrolimus levels between 6 and 8 ng/ml were intended one year after transplantation. In addition, patients received MMF at an oral dose of 1 g twice daily. Steroid-tapering was done according to the protocol, aiming at discontinuation one year after transplantation [26].

### Patient/graft survival and rejection

According to previous definitions, we defined overall kidney graft survival from date of transplantation until patient death, kidney re-transplantation, need for dialysis or loss of follow-up [9]. Accordingly, overall pancreas graft survival was defined as pancreas failure with resumed insulin therapy, patient death or loss of follow-up. We defined overall patient survival from date of transplantation until patient death or loss of follow-up [9].

Delayed graft function (DGF) of the pancreas was defined as the need for insulin substitution at the time of hospital care but without further need after the discharge.

The definition of delayed graft function (DGF) of the kidney is based on range of clinical criteria and there are more than 10 definitions reported in the literature [27–30].

Despite shortfalls, in our study, DGF of the kidney was defined as the need for dialysis at hospital time but without further need after discharge, since it offers the most used standard, by which transplant centers pragmatically report outcomes and which furthermore makes a comparison of published studies on this topic possible.

Acute rejection of the pancreas allograft was defined by an increase of serum lipase and/or amylase, elevated fasting plasma glucose levels, a need for exogenous insulin, a low C-peptide level, an impaired renal function with elevated serum creatinine levels, clinical symptoms (pain, fever, leukocytosis) and/or confirmed by renal histology.

Kidney biopsy was performed when acute rejection of the transplant kidney was clinically suspected. Routine pancreas biopsies were not routinely performed. Rejection episodes were treated with 500 mg of methylprednisolone over three to five days or if steroid-resistant with ATG.

### Measurement of endocrine and metabolic outcome

Endocrine and metabolic function was evaluated at month 1, 6, 12, 24, 36, 48 and 60 months after transplantation. After 8-h fasting, the fasting plasma glucose, HbA1c, C-peptide, low-density lipoprotein (LDL)- cholesterol, high density lipoprotein (HDL) -cholesterol, triglyceride and total cholesterol levels were measured.

### Statistical analysis

Continuous variables were summarized as mean/median values with standard deviation (SD)/minimum or maximum range depending on the normality of the distribution. Categorical variables were expressed as whole numbers and percentages (%). Baseline data were analyzed using the appropriate statistical significance test including a chi-square test, Student’s t–test, analysis of variance (ANOVA), Kruskal-Wallis and/or Wilcoxon–Mann–Whitney test. Survival rates were calculated using the Kaplan-Meier analysis and the log-rank test was applied to test statistical significance. A stepwise Cox proportional hazard regression model and logistic regression was applied for multivariate analysis. All data were analysed by using SPSS software (SPSS Inc., Chicago, Illinois, USA, version 21.0). A *p* value < 0.05 was considered statistically significant.

## Results

Between 2001 and 2013, we included 127 patients with either SPK or KTA allografts into our retrospective study, 101 of whom received SPK and 26 KTA. Twelve of our 101 SPK patients had been prospectively classified as type 2 diabetics according to special request allocation of ET after fulfilling special criteria. The 26 KTA patients served as a control group with diagnosis of T2DM during the same period. Exogenous insulin was administrated in all of our SPK T2DM recipients and in 25 of 26 T2DM recipients with kidney alone transplantation.

### Baseline demographic characteristics

Recipient, donor and pre-transplant baseline characteristics according to diabetes type are summarized in Table 1. The mean follow-up period was 71 +/− 34.4 months.

**Table 1:** Clinicopathologic and demographic characteristics of recipients, donors and transplant compared between T1DM und T2DM

Variables	T1DM SPK (*n* = 89)	T2 DM SPK (*n* = 12)	T2DM KTA (*n* = 26)	*p*-value
Age at onset of diabetes mellitus, years	15.9 +/− 9.1	28.6 +/− 10.9	41.2 +/− 12.4	< 0.01
Recipient age, years	42.3+/− 8.4	48.7+/− 10.6	61.5+/− 8.6	< 0.01
Recipient gender				0.04
Male	49 (55.1%)	8 (66.7%)	21 (80.8%)	
Female	40 (44.9%)	4 (33.3%)	5 (19.2%)	
Recipient BMI (kg/m2)	24.8 +/− 4.1	26.4 +/− 4.9	28.6 +/− 3.1	< 0.01
Duration of Diabetes mellitus, years	27.6 +/− 7.9	18.7 +/− 9.8	18.9 +/− 8.9	< 0.01
Insulin amount, IU/d	47.9 +/− 22.3	37.1 +/− 18.1	42.1 +/− 19.3	n.s.
Donor age, years	23.9 +/− 11.7	17.3 +/− 12.1	59.7 +/− 17.4	< 0.01
Donor BMI (kg/m2)	22.5 +/− 3.5	22.1 +/− 2.6	25.4 +/− 3.5	< 0.01
Donor, gender				n.s.
Male	56 (62.9%)	4 (33.3%)	12 (46.2%)	
Female	33 (37.1%)	8 (66.7%)	14 (53.8%)	
Donor- Cause of Death, CVA %	26 (29.2%)	5 (41.7%)	15 (57.7%)	0.03
CIT Pancreas, hours	10.7 +/− 2.6	10.9 +/− 2.4	N.A.	n.s.
CIT kidney, hours	11.2 +/− 3.2	11.7 +/− 2.8	11.3 +/− 4.9	n.s.
Waiting time, months	7.8 +/− 10.4	15.1 +/− 16.4	22.3 +/− 28.4	0.01
Petransplant dialysis duration, months	31.54 +/−35.19	40.58 +/− 23.3	88.28 +/− 49.1	< 0.01
Pre-emptive transplant	22 (24.7%)	0 (0%)	2 (7.7%)	0.03
Systolic blood pressure, mmHG	134 +/− 17	138 +/− 21	141 +/− 18	0.04
Diastolic blood pressure, mmHG	76 +/− 8	79 +/− 11	82 +/− 11	0.03
HbA1c pretransplantation,%	7.9 +/− 1.7	6.6 +/− 1.4	6.7 +/− 0.9	0.01
C-Peptid, ng/ml	0.15 +/− 0.4	3.2 +/− 1.1	–	< 0.01
Total cholesterol,	5.2 +/− 1.4	5.6 +/− 0.8	4.9 +/− 1.6	n.s.
Triglyceride,	1.9 +/− 1.1	2.5 +/− 1.7	2.6 /− 1.2	0.04
LDL- cholesterol,	2.8 +/− 0.9	2.9 +/− 1.1	2.8 +/− 1.4	n.s.
HDL- cholesterol,	1.5 +/− 0.4	1.2 +/− 0.3	1.2 +/− 0.4	0.02
Arterial obstructive disease				0.04
Yes	15 (16.9%)	2 (16.7%)	10 (40%)	
No	74 (83.1%)	10 (83.3%)	15 (60%)	
Coronary heart disease				< 0.01
Yes	23 (25.8%)	6 (50%)	19 (73.1%)	
No	66 (74.2%)	6 (50%)	7 (26.9%)	
CABG/stent				< 0.01
Yes	15 (83.1%)	4 (33.3%)	16 (64%)	
No	74 (16.9%)	8 (66.7%)	9 (36%)	
Retinopathy				< 0.01
Yes	78 (87.6%)	8 (66.7%)	10 (38.5%)	
No	11 (12.4%)	4 (33.3%)	16 (61.5%)	
Neuropathy				n.s.
Yes	55 (73.3%)	9 (75%)	12 (46.2%)	
No	34 (38.9%)	3 (25%)	14 (53.8%)	
Nephropathy				N.A.
Yes	89 (100%)	12 (100%)	26 (100%)	
No	0 (0%)	0 (0%)	0 (0%)	
Number of antihypertensive drugs				n.s.
0	14 (15.7%)	3 (25%)	9 (34.6%)	
1	6 (6.7%)	1 (8.3%)	2 (7.7%)	
2	14 (15.7%)	2 (16.7%)	6 (23.1%)	
3	24 (27%)	2 (16.7%)	5 (19.2%)	
4	18 (20.2%)	1 (8.3%)	2 (7.7%)	
> 4	13 (14.6%)	3 (25%)	2 (7.7%)	
Panel reactive antibodies (PRA), %	75 (85.2%)	12 (100%)	19 (73.1%)	n.s.
0	10 (11.4%	0	5 (19.2%)	
1–20 > 20	3 (3.4%)	0	2 (7.7%)	
HLA-DR mismatch; n	1.3 +/− 0.5	1.1 +/− 0.4	2.3 +/− 1.4	< 0.01
CNI				n.s.
Tacrolimus	85 (95.5%)	12 (100%)	25 (96.2%)	
Cyclosporin	4 (4.5%)	0 (0%)	1 (4.5%)	
Induction Therapy				< 0.01
ALG/ATG	63 (70.8%)	11 (91.7%)	4 (15.4%)	
IL2-RA	19 (21.3%)	0 (0%)	12 (46.2%)	
None	7 (7.9%)	1 (8.3%)	10 (38.5%)	
AP drug				0.01
MMF	72 (80.9%)	11 (91.7%)	22 (84.6%)	
SRL	13 (14.6%)	1 (8.3%)	0	
Multiple	3 (3.4%)	0	0	
NONE	1 (0.8%)	0	4 (15.4%)	

T1DM recipients were older at their time of diabetes onset (*p < 0.01*) and at the time of transplant (*p < 0.01*). Furthermore, they had a higher BMI (*p < 0.01*), were more male (*p = 0.04*) and had a higher pre-transplant dialysis duration time (*p < 0.01*) than T2DM patients.

In contrast T2DM recipients had fewer years of diabetes disease (*p < 0.01*).

Regarding donor characteristics, the donor age in the T1DM SPK recipients` group was higher (*p < 0.01*) and in parallel the BMI higher too (*p < 0.01*). Other significant differences were a shorter waiting time (*p < 0.01*) and a higher rate of pre-emptive transplantations (*p < 0.01*) in the T1DM recipients group. In type I and II diabetic recipients, the average pre-transplant insulin dose was 47.9 +/− 22.3 and 37.1. +/− 18.1 IU/d for SPK transplant recipients, respectively.

Furthermore, a statistically significant difference in pre-transplant comorbidities was detected with regard to secondary diabetes complications (retinopathy), peripheral arterial obstructive and coronary heart disease with coronary interventions, HLA mismatches, induction therapy agents and AP drugs, blood pressure, endocrine (C-peptid, HbA1c) and lipid metabolism (triglyceride, HDL- cholesterol) between groups.

### Peri- and Posttransplant complications

Peri- and posttransplant complications in T1DM and T2DM recipients are shown in Table 2. There were no significant differences in the rate of peri- and posttransplant complications including infections, rejections, delayed graft function of the pancreas, surgical complications and C-peptid levels at discharge. In the T2DM KTA group, delayed graft function was significantly more frequent compared to SPK recipients (*p* = 0.04) and KTA recipients had significantly higher levels of serum creatinine level at discharge higher (*p < 0.01*). Pancreas associated complications were similar between both groups.

**Table 2:** Peri- and post transplant clinical data of the study group

Variables	T1DM SPK (*n* = 89)	T2 DM SPK (*n* = 12)	T2DM KTA (*n* = 26)	*p*-value
One-year cumulative combined kidney and pancreas Rejection				
Yes	19 (21.3%)	7 (58.3%)	4 (15.4%)	n.s.
No	70 (78.7.%)	5 (41.7%)	22 (84.6%)	
DGF Pancreas			N.A.	n.s.
Yes	4 (4.5%)	1 (8.3%)		
No	85 (95.5%)	11 (91.7%)		
DGF kidney				0.04
Yes	13 (14.9%)	2 (16.7%)	11 (42.3%)	
No	74 (85.1%)	10 (83.3%)	15 (57.7%)	
Infectious Complications				n.s.
Yes	18 (20.2%)	4 (33.3%)	10 (38.4%)	
No	71 (79.8%)	8 (66.7%)	16 (61.6%)	
Pancreas Complications				
Graft Thrombosis				
Yes	9 (10%)	1 (9.1%)	N.A.	n.s.
No	79 (90%)	11 (90.9%)		
Abscess/local Infection				
Yes	6 (6.7%)	1 (8.3%)	N.A.	n.s.
No	83 (93.3%)	11 (91.7%)		
Anastomotic leak				
Yes	1 (1.1%)	0	N.A.	n.s.
No	0 (98.9%)	0		
Pancreatitis				
Yes	11 (12.2%)	2 (18.2%)	N.A.	n.s.
No	79 (78.2%)	10 (81.8%)		
Bleeding				
Yes	8 (9%)	2 (16.7%)	N.A.	n.s.
No	81 (91%)	10 (83.3%)		
C-peptide at discharge, mean (SD), ng/ml	2.28 (1.8)	3.19 (1.79)		n.s.
Creatinine at discharge, mean (SD), ummol/l	130.5+/−88.1	152.1+/−74.7	240.3+/−130.1	< 0.01

### Metabolic outcome for T1DM und T2DM SPK transplant recipients

**Fig. 1:**
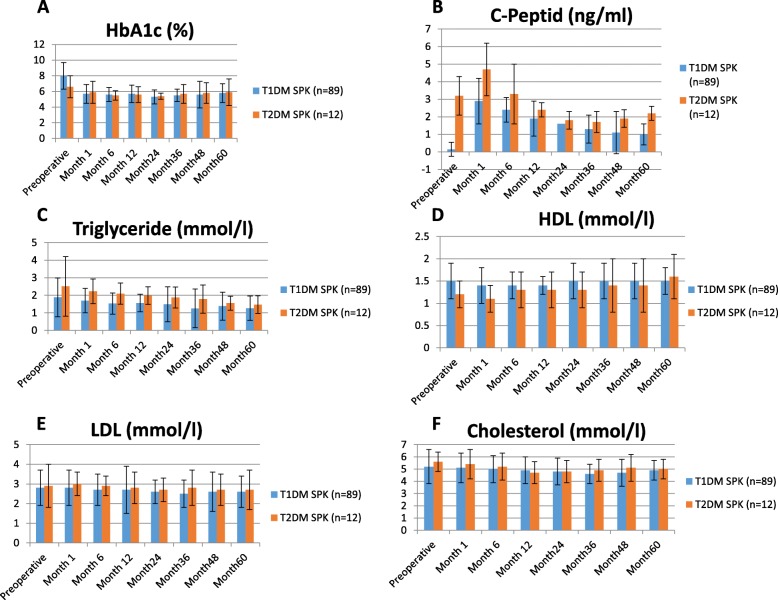
Endocrine and metabolic outcome of the Study Group. The mean level of HbA1c **a**, C-peptide **b**, triglyceride **c**, HDL- cholesterol **d**, LDL- cholesterol **e**, total cholesterol **f** according to the type of diabetes in SPK patients until postoperative 5 years

The mean level of HbA1c was significantly lower in T2DM SPK transplant recipients in comparison to T1DM recipients before SPK (*p = 0.02*) (Table 1**;** Fig. 1a). However, after SPK there were no significant differences in the mean HbA1c levels between both groups over the follow up period, and the levels remained constant below 6% until 5 years post-transplant (Fig. 1a). The level of C-peptide was significantly higher in the T2DM recipients compared with T1DM before SPK transplantation (*p < 0.01*) (Fig. 1b). The postoperative mean levels of C-peptide in T2DM recipients were also significantly higher than those in T1DM recipients during 5 year follow up period (*p = 0.01*). However, the levels decreased steadily which was confirmed by a linear mixed effect model (*p = 0.03*). No significant differences were observed in fasting glucose levels, tacrolimus levels, BMI such as blood pressure values at 1, 6, 12, 24, 36, 48 and 60 months after transplantation between T1DM und T2DM SPK transplant recipients.

Regarding lipid profiles, significant differences were observed in HDL- cholesterol and triglyceride levels between both groups. The mean triglyceride level was significantly lower in T1DM recipients compared to T2DM before transplantation (*p = 0.04*) (Fig. 1c). However, mean HDL- cholesterol levels were significantly higher in T1DM recipients compared to T2DM patients before transplantation (T1DM 1.5 +/− 0.4 mmol/l versus 1.2+/− 0.3 mmol/l for T2DM, *p = 0.02*) and remained significantly over the follow up period 5 years after transplantation (*p = 0.04*) (Fig. 1d).

LDL- cholesterol and total cholesterol levels were similar between both groups over the observed follow up period (Fig. 1e and f).

In the early follow-up period (3 months) after transplantation, there were significant differences in creatinine levels between the SPK group (T1DM: 123 +/− 72 umol/l versus T2DM: 152 +/− 82 ummol/l) and the KTA group (175 +/− 79 ummol/l) (*p < 0.01*). However, the creatinine levels remained stable without significant differences between the three groups at follow-up 5 years after transplantation (SPK group: T1DM: 135 +/− 88 ummol/l versus T2DM: 133+/− 21 ummol/l; KTA group: 195 +/− 72 ummol/l) (*p = 0.30*).

### Patient, kidney and pancreas survival

**Fig. 2:**
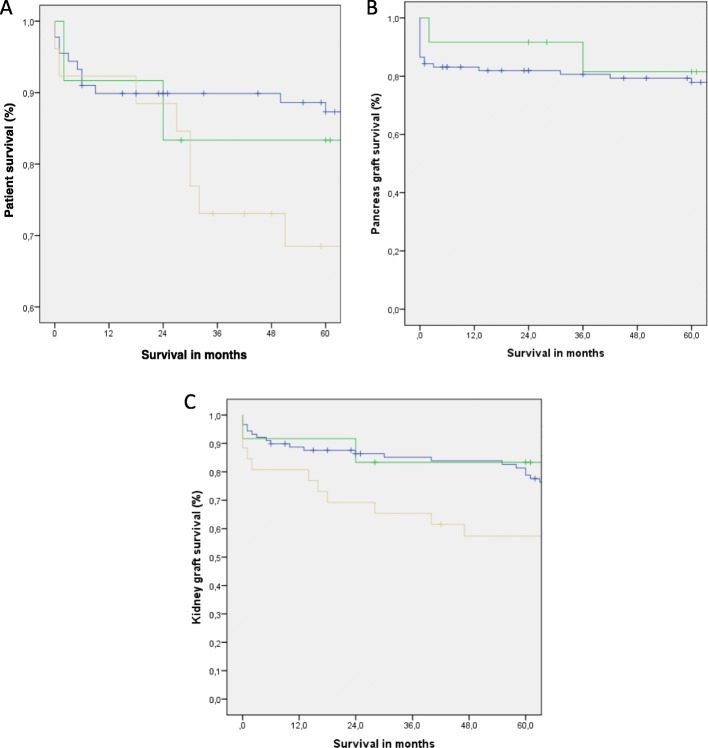
Patient survival and pancreas and kidney graft survival according to the T1DM SPK, T2DM SPK and T2DM KTA groups

Patient, pancreas and kidney graft survival 1,3 and 5 years after transplantation is shown in Table 3, Table 4 and Fig. 2.

**Table 3:** Cox regressions analysis for independent factors associated with SPK transplant outcomes

Variables	Reference	Levels	Univariate Analysis		Multivariate Analysis	
			HR (95%CI)	*p*-value	HR (95%CI)	*p*-value
Patient death						
Diabetes	T1DM	T2DM	1.35 (0.23–6.01)	0.70		
Recipient age	< 45 years	> 45 years	3.2 (1.1–8.7)	0.02	2.4 (0.9–6.1)	0.07
Recipient Gender	Male	Female	0.5 (0.15–1.37)	0.16		
Coronary heart disease	No	Yes	3.15 (1.06–9.38)	0.04		
Arterial obstructive disease	No	Yes	3.26 (1.1–9.99)	0.04		
Pretransplant dialysis	Pre-emptive	> 1 year	2.9 (1.2–7.2)	< 0.01	4.5 (1.5–15.2)	0.01
Donor age	< 45 years	> 45 years	2.3 (0.8–6.1)	0.02	1.3 (1.1–5.1)	0.04
BMI recipient	< 25	> 25	2.23 (0.67–7.5)	0.19		
Donor COD	No CVD	CVD	1.1 (0.3–2.7)	0.98		
Calcineurininhibitor	CNI	Tacrolimus	0.41 (0.5–3.2)	0.38		
Donor gender	Male	Female	3.98 (0.88–17.92)	0.07		
Donor BMI	< 25 kg/m2	> 25 kg/m2	2.29 (0.68–7.77)	0.18		
Pancreas Graft Loss						
Diabetes	T1DM	T2DM	1.34 (0.6–2.6)	0.46		
Recipient age	< 45 years	> 45 years	5.38 (1.99–14.51)	< 0.01	3.89 (1.40–10.78)	0.01
Coronary heart disease	No	Yes	1.21 (0.50–2.94)	0.68		
Arterial obstructive Disease	No	Yes	1.48 (0.51–3.72)	0.44		
Pretransplant dialysis	Pre-emptive	> 1 year	1.19 (0.42–3.44)	0.74		
Donor age	< 45 years	> 45 years	2.69 (0.80–9.1)	0.11		
Recipient BMI	< 25	> 25	4.24 (1.57–11.43)	< 0.01	3.40 (1.21–9.59)	0.02
CIT Pancreas	< 12 h	> 12 h	2.62 (1.13–6.07)	0.03	3.25 (1.25–8.45)	0.02
HLA-Mismatch	0	5 to 6	1.1 (1.0–3.2)	< 0.01	1.3 (1.1–5.2)	< 0.01
Infectious complications	No	Yes	1.3 (0.4–3.9)	0.06		
Surgical complications	No	Yes	5.9 (1.8–19.4)	< 0.01	4.8 (2.3–11.6)	< 0.01
Recipient gender	Male	Female	0.55 (0.24–1.24)	0.15		
Donor gender	Male	Female	1.36 (0.58–3.22)	0.48		
Induction Therapy	None	ATG	0.6 (0.3–1.5)	0.38		
Donor BMI	< 25	> 25	4.89 (2.1–11.45)	< 0.01	3.59 (1.45–8.92)	0.01
Kidney Graft Loss						
Diabetes	T1DM	T2DM	1.1 (0.3–3.1))	0.95		
Recipient age	< 45 years	> 45 years	1.2 (0.5–2.8)	0.62		
Coronary heart disease	No	Yes	2.1 (0.3–1.6)	0.19		
Arterial obstructive disease	No	Yes	2.2 (0.6–5.6)	0.19		
Pretransplant dialysis	Pre-emptive	> 1 year	1.1 (0.3–1.9)	< 0.01	1.2 (1.1–1.6)	< 0.01
Donor age	< 45 years	> 45 years	1.8 (0.8–6.8)	0.02	2.3 (0.5–7.2)	0.05
BMI Recipient	< 25	> 25	1.3 (0.4–2.9)	0.70		
HLA Mismatch	0	5to6	1.2 (1.0–3.2)	< 0.01	1.4 (1.2–6.2)	< 0.01
CIT Kidney	< 11 h	> 11 h	1.7 (0.8–3.66)	0.16		
Recipient gender	Male	Female	0.72 (0.3–1.4)	0.43		
Infectious complications	No	Yes	1.4 (0.6–3.4)	0.39		
Rejection	No	Yes	1.3 (0.6–2.7)	0.47		
Donor BMI	< 25	> 25	0.91 (0.3–2.4)	0.85		
Donor COD	Others	CVD	1.1 (0.5–2.5)	0.80		
DGF Kidney	No	Yes	1.8 (0.6–5.2)	0.26		
Surgical complications	No	Yes	1.9 (0.7–2.1)	0.08		
Induction Therapy	None	ATG	1.1 (0.5–2.5)	0.80

**Table 4:** Patient survival and pancreas and kidney graft survival according to the T1DM SPK, T2DM SPK and T2DM KTA groups

A: Patient survival according to the T1DM SPK T2DM SPK and T2DM KTA groups.				
*Patient Survival*	*T1DM SPK*	*T2DM SPK*	*T2DM KTA*	*p-value*
*1-y*	*89.9%*	*91.7%*	*92.3%*	*< 0.01*
*3-y*	*89.9%*	*83.3%*	*73.1%*	
*5-y*	*88.8%*	*83.3%*	*69.2%*	
B: Pancreas Graft survival according to the T1DM SPK T2DM SPK and T2DM KTA groups.				
Pancreas Graft	T1DM SPK	T2DM SPK	*p*-value	
Survival				
1-y	83.1%	91.7%	0.71	
3-y	79.8%	83.3%		
5-y	78.7%	83.3%		
C: Kidney graft survival according to the T1DM SPK T2DM SPK and T2DM KTA groups.				
Kidney Graft	T1DM SPK	T2DM SPK	T2DM KTA	*p*-value
survival				
1-y	88.8%	91.7%	80.8%	< 0.01
3-y	85.4%	83.3%	65.4%	
5-y	79.8%	83.3%	57.7%

Patient survival (Fig. 1a) at 1, 3 and 5 years was significantly higher in T1DM recipients as compared with the T2DM groups (*p < 0.01*) (Fig. 2a).

Overall pancreas graft survival was better at all three time points in the T2DM SPK group but did not reach statistical significance (Fig. 2b).

Overall kidney graft survival (5 year kidney graft survival: 79.8% for T1DM SPK versus 83.3% for T2DM SPK and 57.7% for T2DM KTA; *p < 0.01*) was significantly inferior in T2DM recipients after kidney transplantation alone (Fig. 2c).

However, the type of diabetes mellitus could not be identified as a risk factor for outcomes in uni- and multivariate analyses. Hazard ratios were 1.35 (95%CI: 0.23–6.01) for patient death, 1.34 (0.6–2.6) for overall pancreas graft failure and 1.1 (95%CI: 0.1–3.1) for overall kidney graft failure in T2DM recipients, compared with T1DM as the reference group.

Risk factors independently associated with outcomes in the overall SPK cohort were shown in Table 3.

Following factors could be found as significant parameters for death in univariate analysis: recipient age > 45 years (versus < 45 years, *p = 0.02*), donor age > 45 years (versus < 45 years, *p = 0.02*), > 1 year pretransplant dialysis (versus preemptive; *p < 0.01*), comorbidities such as coronary heart disease (versus no disease, *p = 0.04*) and peripheral arterial obstructive disease (versus no disease, *p = 0.04)*.

After entering these parameters in a multivariate COX regression analysis, following factors remained significant for death: donor age > 45 years versus < 45 years (HR 1.3 (CI: 1.1–5.1); *p = 0.04*) and pre-emptive transplantation versus dialysis > 1 year (HR 4.5 (CI: 1.5–15.2); *p = 0.01*).

Significant recipient, donor and transplant characteristics associated with pancreas graft failure were recipient age > 45 years versus < 45 years (HR 3.89 (CI: 1.4–10.8); *p < 0.01),* recipient BMI > 25 kg/m2 versus < 25 kg/m2 (HR 3.4 (CI: 1.21–9.59); *p = 0.02),* donor BMI > 25 kg/m2 versus < 25 kg/m2 (HR 3.59 (CI: 1.45–8.92); *p < 0.01),* cold ischemia time of the pancreas > 12 h versus < 12 h (HR 3.25 (CI: 1.25–8.45); *p = 0.02*) and surgical complications (HR 4.8 (CI: 2.3–11.6); *p < 0.01*).

Whereas, a dialysis duration > 1 years versus preemptive transplantation (HR 1.2 (CI: 1.1–1.6); *p < 0.01*) and HLA-mismatch 5 to 6 versus 0 (HR 1.4 (CI: 1.2–6.2); *p < 0.01*) were associated with increased risk for kidney allograft failure. Donors > 45 years versus < 45 years (HR 2.3 (CI: 0.5–7.2); *p = 0.05*) and surgical complications (yes versus no; HR 1.9 (CI: 0.7–2.1), *p = 0.08)* had a trend due to increased kidney allograft failure.

## Discussion

Is SPK transplantation nowadays still suitable for T2DM patients? The answer to this question is still pending. However, our data reinforce the fact that a selected group of T2DM patients significantly benefit from SPK.

Improved success rates, favorable risk-benefit ratios and novel immunosuppressive therapies developed over the last decades definitely made pancreas transplantation a story of success, not only for T1DM but also for T2DM patients, and those with brittle pancreaticogenic diabetes. Today the efficacy of SPK especially in selected T2DM, C-peptide positive patients with end stage renal disease is well accepted. However, the current literature does not provide prospective randomized trials on SPK for this set of patients and as a limitation our study also does not address this need. In an initial report in the year 2005, Light et al. described their experiences of 135 insulin-dependent patients with ERDS undergoing SPK for either T1DM or T2DM. The groups were defined by the level of C-peptide with a cut-off point of 0.8 ng/ml. In their 10-year follow up, patient and graft survival were similar although groups differed significantly in terms of age, BMI and ethnicity [12].

A subsequent analysis by Singh et al. used higher C-peptide cut-off levels (2.0 ng/ml) for the better discrimination of T1DM and T2DM patients [13]. As expected, in this study patients with higher C peptide levels were older, had a higher BMI and a later onset and shorter duration of diabetes mellitus, as well as a longer duration of pre-transplant dialysis. And again, death censored kidney and pancreas graft survival rates were similar for both groups. These early studies demonstrate that comparable outcomes can be achieved for PTX in T1DM and T2DM patients.

Our T2DM patients listed for SPK displayed accordingly to the ET listing criteria (which for the most part resemble the guidelines of the American Diabetes Association (ADA) and World Health Organization (WHO)) a maximum bodyweight no greater than 115% of the ideal body weight, which reflects a BMI < 30 kg/m^2^ [6, 7, 17]. Furthermore, a pronounced metabolic syndrome was not present at time of transplantation, since these patients with no doubt might rather benefit from a bariatric surgical intervention than from transplantation [31, 32].

End stage renal disease (ESRD) is a serious development in diabetes mellitus and represents a serious clinical problem which lacks effective therapy for the last 20 years. A great body of evidence supports the fact that C-peptide has a beneficial effect on disturbed physiologic pathways which lead to the development of diabetic nephropathy and short-term studies of C-peptide therapy in patients with ESRD have indicated beneficial effects such as lowered hyperfiltration rate and reduced albuminuria [33].

Peri- and post- SPK transplant complication rates as well as pancreas associated complication rates described here were similar for both groups (T1DM and T2DM) as described earlier [9–14, 34]. The higher delayed graft function (DGF) rate and serum creatinine level at discharge in our T2DM control group which only received a KTA may be attributed to an inferior donor organ quality as reflected in a higher donor age and BMI [35]. Comparisons of patient groups are complicated due to differences in SPKT and KTA recipients as well as different prioritizations on the waiting list for both patient groups. The SPKT patient by nature, has fewer comorbidities, is younger and predominantly suffers from T1DM. The small group of T2DM patients who qualify for SPKT by law do not have a profound metabolic syndrome and benefit from shorter waiting time and superior organ quality, since combined pancreas and kidney allografts categorically originate from young non marginal donors. With no doubt, both groups must be considered to more likely survive long term than the typical diabetic KTA patient.

From this point of view conclusions that superior outcomes for SPKT recipients may be solely attributed to the pancreas transplant or the type of patient that receives a SPKT must be seen critically. Hence, most analyses including our own, that compare SPKT and KTA patients, conclude that the benefit of the pancreas transplant is modest [36–38].

The primary indication for pancreas transplantation worldwide remains T1DM, and still many US and European centers consider T2DM as a contraindication for transplantation [5, 39, 40]. On the other hand, T2DM which is in parallel one of the leading causes of kidney disease, appears to have an increasing prevalence in most western countries [41]. However, the explicit differential-diagnosis between T1DM and T2DM often remains inconclusive, since obesity and later age of onset often mask the distinct disease characteristics.

Previous studies focused on the long-term metabolic functions after pancreas transplantation, but they were predominantly confined to T1DM patients receiving a SPK [18, 20, 42]. Although several studies have described favorable long-term outcomes of pancreas transplantation in Patients with T2DM little information on the metabolic outcome is sparse [9, 11, 14].

Despite our T2DM recipients were older, with a higher BMI and a longer pre-transplant dialysis duration, endocrine and metabolic short- and long- term function after SPK transplantation showed consistent good results over the entire observation period. And beyond dispute, a survival advantage of SPKT over KTA may be due to superior organ availability and shorter waiting times [43].

Most transplant groups including our own recommend avoiding patients with evidence of significant metabolic syndrome and demand a patient specific approach to the T2DM transplant candidate [7, 15].

## Conclusion

Taken together, there is a small group of T2DM patients who benefit from SPK and both short- and long-term results are comparable to T1DM patients receiving a transplant. Further studies and the future implementation of consensus guidelines for T2DM receiving a SPK might be beneficial.

## Data Availability

The datasets generated and/or analyzed during the current study are not publically available because our database contains highly sensible data which may provide insight in clinical and personnel information about our patients and lead to identification of these. Therefore, according to organizational restrictions and regulations these data cannot be made publically available. However, the datasets are available from the corresponding author on reasonable request.

## Abbreviations

ADA: American Diabetes Association
ALG: Anti-lymphocyte globulin
ANOVA: Analysis of variance
AP: Antiproliferative
ATG: Anti-thymocyte globulin
BMI: Body mass index
CABG: Coronary artery bypass graft
CIT: Cold ischemia time
CNI: Calcineurin inhibitors
COD: Cause of death
CVA: Cerebrovascular accident
DGF: Delayed graft function
ESRD: End-stage renal disease
ET: Eurotransplant
HDL: High-density lipoprotein
HLA: Human leukocyte antigen
HR: Hazard Ratio
IL2-RA: Interleukin-2 receptor antagonist
KTA: Kidney transplantation alone
LDL: Low-density lipoprotein
MMF: Mycophenolate mofetil
NA: Not applicable
PRA: Panel reactive antibodies
SD: Standard deviation
SPK: Simultaneous pancreas-kidney
SRL: Sirolimus
T1DM: Type 1 diabetes mellitus
T2DM: Type 2 diabetes mellitus
WHO: World Health Organization
